# Effect of n-3 polyunsaturated fatty acid on gene expression of the critical enzymes involved in homocysteine metabolism

**DOI:** 10.1186/1475-2891-11-6

**Published:** 2012-01-19

**Authors:** Tao Huang, Mark L Wahlqvist, Duo Li

**Affiliations:** 1 Department of Food Science and Nutrition, Zhejiang University, Hangzhou, 310029 China; 2 APCNS Centre of Nutrition and Food Safety, Hangzhou, China; 3 National Health Research Institutes, Zhunan, Taiwan

## Abstract

**Background:**

Previous studies showed that plasma n-3 polyunsaturated fatty acid (PUFA) was negatively associated with plasma homocysteine (Hcy).

**Objective:**

We investigated the regulatory effect of n-3 PUFA on mRNA expression of the critical genes encoding the enzymes involved in Hcy metabolism.

**Methods:**

HepG2 cells were treated with docosahexaenoic acid (DHA), eicosapentaenoic acid (EPA), alpha-linolenic acid (ALA) respectively for 48 h. The cells were collected and total RNA was isolated. The mRNA expression levels of the genes were determined by using Real Time-PCR.

**Results:**

Compared with controls, the mRNA expression levels of 5-methyltetrahydrofolate reductase (MTHFR) were significantly increased in the DHA group (p < 0.05) and ALA group (p < 0.05); Significantly down-regulated mRNA expression of methionine adenosyltransferase (MAT) was observed with the treatments compared with the controls; the level of MAT expression was significant lower in the DHA group than the ALA group (p < 0.05); Cystathionine-γ-lyase (CSE) expression was significantly increased in the DHA (p < 0.05) and EPA groups (p < 0.05) compared with control. No significant changes were shown in mRNA expression levels of S-adenosylhomocysteine hydrolases (SAHH), cystathionine β-synthase (CBS), and 5-methyltetrahydrofolate-homocysteine methyltransferase (MTR).

**Conclusions:**

Our results suggest that DHA up-regulates CSE and MTHFR mRNA expression and down-regulates MAT mRNA expression involved in Hcy metabolism.

## Background

**Figure 1:**
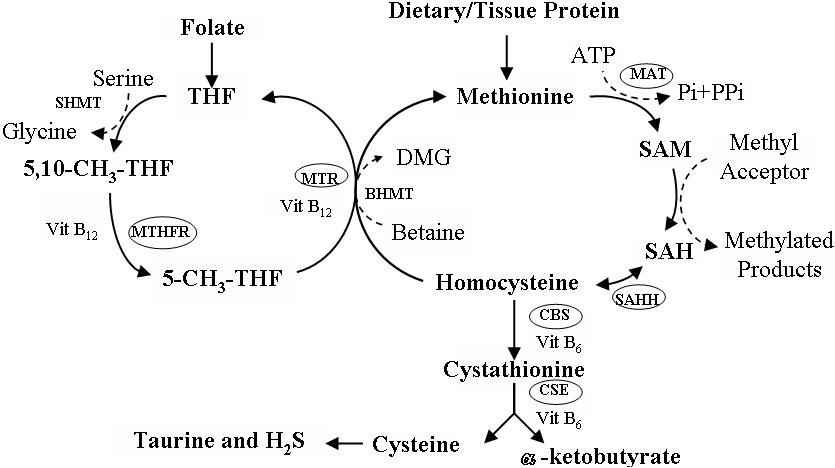
**The diagram shows the enzyme points (in cycle) on which the effects of n-3 PUFA might operate in the homocysteine metabolic pathway of relevance**. The critical genes in cycle were determined in present study. MTR: 5-methyltetrahydrofolate-homocysteine methyltransferase; MAT: methionine adenosyl transferase; SAHH: S-adenosylhomocysteine hydrolases; CBS: Cystathionine β-synthase; CSE: Cystathionine γ-lyase; MTHFR: Methylenetetrahydrafolate reductase; BHMT: Betaine-homocysteine methyltransferase; DMG: Dimethylglycine; 5, 10-CH_3_-THF: 5, 10-Methylene-Tetrahydrofolate; 5-CH_3_-THF: 5-Methyl-Tetrahydrofolate; THF: Tetrahydrofolate; SHMT: Serine hydroxymethyl transferase; Pi: Orthophosphate; PPi: Pyrophosphate.

Hyperhomocysteinaemia (HHcy) has been reported to be an independent risk factor for cardiovascular disease (CVD) [1]. Homocysteine (Hcy) is a thiol-containing amino acid derived from methionine metabolism [2]. In methionine metabolism, methionine is converted to S-adenosylmethionine (SAM) via methionine adenosyltransferase (MAT), which is the only methyl-donating pathway in humans [3]. S-adenosylhomocysteine (SAH), a product of this methyl-transferase reaction, is hydrolyzed to Hcy in a reversible reaction via the S-adenosylhomocysteine hydrolases (SAHH). Once synthesized, Hcy can be degraded through two enzymatic pathways: transsulfuration and remethylation (Figure 1) [1]. In remethylation, Hcy can be converted back to methionine in the remethylation pathway via 5-methyltetrahydrofolate reductase (MTHFR) and methionine synthase (MS) using cofactors such as vitamin B_12 _and folic acid [4]. In the transsulfuration pathway, Hcy is condensed with serine to form cystathionine via vitamin B_6 _dependent cystathionine β-synthase (CBS), subsequently, cystathionine is converted to cysteine, α-ketosuccinic acid, taurine, and hydrogen sulfide (H_2_S) via vitamin B_6 _dependent cystathionine-γ-lyase (CSE) [5].

Variations in the levels of Hcy can be due to defects of the genes encoding the critical enzymes involved in methionine metabolism [6], nutritional status for folic acid, vitamin B_6 _and B_12_, and various personal behaviours like physical inactivity and smoking [7]. Hcy metabolism is nutritionally regulated in part through the utilization of Hcy in the transsulfuration and remethylation pathways [8].

N-3 polyunsaturated fatty acids (PUFA) have protective effects on the cardiovascular system. Dietary intake of fish oil rich in n-3 PUFA leads to increased levels of n-3 PUFA in tissues [9], which is associated with reduced incidence of cardiovascular events via regulatory effects on blood pressure [10], serum/plasma triacylglycerol (TG) levels [11], antithrombotic effects and heart rate variability [12].

In a previous study, we reported that plasma Hcy was also significantly and negatively correlated with plasma phospholipids (PL) 22:6n-3 (DHA), total n-3 PUFA and n-3/n-6 PUFA in healthy Australian men [13], and in middle aged and geriatric hyperlipaemic patients in Hangzhou [14]. In our cross sectional study, *MAT1A *genotypes were found to interact with dietary PUFA in determining plasma Hcy [8]. Furthermore, intervention studies and our recent meta-analysis document that the high consumption of n-3 PUFA decreases plasma Hcy [15]. However, the mechanisms which may explain the relationship between n-3 PUFA and plasma Hcy levels are not yet fully understood. In a previous report we suggested that a possible mechanism for this relationship is that n-3 PUFA, especially 22:6n-3, may modulate gene expression of an enzyme involved in the formation and metabolism of plasma Hcy [13].

Furthermore, in mammals, two genes (MAT1A and MAT2A), play an important role in human hepatocellular carcinoma, through facilitation of cancer cell growth [16]. In addition, SAH and Hcy is associated with invasion activities of hepatoma cells, increased level of plasma Hcy is a reflection of the degree of liver injury is more sensitive biochemical indicator of liver cirrhosis and liver cancer [17]. Therefore, to investigate the potential mechanism by which n-3 PUFA decrease Hcy in HepG-2 cell line, we conducted the cell culture to examine the nutritional regulation of n-3 PUFA (22:6n-3, DHA; 20:5n-3, EPA; 18:3n-3, ALA) on the mRNA expression of the genes encoding the key enzymes involved in Hcy metabolism.

## Methods

### Cell Culture

HepG-2 cell was purchased from Chinese Academy of Science. HepG-2 was cultured in DMEM medium (Inalco, USA) supplemented with 10% fetal bovine serum at 37°C in a humidified atmosphere of 5% CO_2_. Cells were seeded in 25-cm^2 ^cell culture flasks or in 12-wells plastic plate (Corning, USA), and grown to 50-70% confluence.

### Treatment of fatty acid on HepG2 cells

Prior to experiments, cells were washed twice with phosphate buffered saline (PBS) and once with serum-free DMEM medium without antibiotics. One milliliter containing 1 × 10^5 ^HepG2 cells was added into each well of the 12-wells plastic plate. For fatty acid treatment, the fatty acids were dissolved in ethanol [18]. After incubation at 37°C for 24 h, the treatment groups were added with fresh culture media with a final fatty acids concentration (DHA, EPA, ALA, Cayman, USA) of 150 μM [19], Controls were exposed to an equal concentration of ethanol to that in the fatty acid exposed samples. After the treatment with the fatty acids for 48 h, the cells were collected for further experiments. Total RNA was extracted with Trizol reagent (Shinegene, China) as described by Zhang et al [20]. RNA was quantified with Nanodrop (Peqlab, Erlangen, Germany) and the RNA integrity number (RIN) was measured with Bioanalyzer (Agilent, Böblingen, Germany). No RNA was used with a RIN below 8.5. All experiments were performed in triplicate and repeated at least three times.

### Assay of the fatty acid composition in HepG2 cell membrane

The cell was collected and centrifuged at 800 rcf. Total lipid content of cell membrane was extracted with chloroform/methanol solution (2:1, vol/vol) containing 50 mg/L butylated hydroxytoluene (Tokyo Kasei Kogyo Co., Ltd., Tokyo, Japan)., The PL fraction was separated by thin layer chromatography (TLC). The PL fatty acids were converted to methyl ester by using 2 mL of 2% H_2_SO_4_-methanol for 2 hours at 70°C. The fatty acid methyl esters were prepared and separated by gas-liquid chromatography as described previously [21].

### Assay of mRNA expression of the critical genes involved in methionine metabolism

#### Real-time polymerase chain reaction

Total RNA from ethanol or n-3 PUFA treated HepG2 cells were extracted by using the Trizol reagent (Shingene, Shanghai, China). The mRNA concentration and mRNA quality were determined by using the NanoDrop ND-2000. A value of 260/280 ratio (1.8-2.0) indicates that the RNA is pure. The first strand cDNA was synthesized using cDNA sythesis kit (shinegene, Shanghai, China), the Real Time-PCR was conducted on iCycler PCR using the HotStart DNA Master SYBR Green I kit (Takara, Dalian, China) [22]. The primers used for the variety genes studied are shown in Table 1. All PCR tests were carried out in duplicate with a final volume of 20 μL containing cDNA. The thermal cycling conditions used were as follows: an initial DNA denaturation step at 95°C for 5 seconds, followed by 40 cycles of denaturation at 95°C for 5 seconds, primer annealing at optimal temperature for 20 s, extension at 72°C for 30 s, and an additional incubation step at 80-85°C for 30 s to measure SYBR Green I fluorescence. Finally, melt curve analysis was performed by slowly cooling the PCR from 95 to 60°C (0.5°C per cycle) with simultaneous measurement of the SYBR Green I signal intensity.

**Table 1:** The primers used in the Real Time-PCR.

Genes	GenBank accession number	Primers
**MAT**	NM_013283	F: 5'- ACTTTGTTCCCGGGAGCTGTC -3'
		R: 5'- AACTGCATGCCAATTATTCTGCTG -3'
**SAHH**	NM_000687	F: 5'- CACCACAGGCTGTATTGACATCATC -3'
		R: 5'- GTCCAATGTTACACACAATGGCATC -3'
**MTR**	NM_000254	F: 5'-TAAGATTTGCAAAGGTTGGGTCTGA-3'
		R: 5'-CTGGACATACAGGTGGGAGTTGG-3'
**MTHFR**	NM_005957	F: 5'- TGTGTGAATTCTGCAACTAGCCAAG -3'
		R: 5'- ATGAGCCACCACACCTGCTG -3'
**CSE**	NM_153742	F: 5'-CCTTTGGCTCTGGGAGCTGATA-3'
		R: 5'-ATTAACAGACACCAGGCCCATTACA-3'
**CBS**	NM_000071	F: 5'-TGTGGGCACACCATCGAGA-3'
		R: 5'-AGCGTCACCATTCCCAGGATTA-3'
**β-actin**	NM_001101	F: 5'- CGACAACGGCTCCGGCATGT-3'
		R: 5'- TGGGCCTCGTCGCCCACATA-3'

**MAT**: methionine adenosyltransferase; **SAHH: ***S*-adenosylhomocysteine hydrolase; **MTR: **5-methyltetrahydrofolate-homocysteine methyltransferase; **MTHFR: **5, 10-methylenetetrahydrofolate reductase; **CSE: **cystathionine γ-lyase; **CBS: **cystathionine β-synthase; **β-action: **housekeeping gene

#### The quantification of Real Time-PCR

Gene expression was quantitated by using the comparative C (t) method [23].

C (t), the threshold cycle, is the number of cycles it takes for a sample to reach the level where the rate of amplification is the greatest during the exponential phase.

The quantification is used to determine the ratio between the quantity of a target molecule in a sample and in the calibrator (calibrator being e.g. untreated cell). The most common application of this method is the analysis of gene expression, e.g. comparisons of gene expression levels in different samples. Target molecule quantity is usually normalized with a housekeeping gene. Comparative C (t) method can be used for relative quantification. Both the sample and calibrator data is first normalized against variation in sample quality and quantity. Normalized values, C (t) s, are first calculated from following equations:

$$\begin{array}{c} \Delta \text{C }{\left(\text{t}\right)}_{\text{sample}}=\text{ C }{\left(\text{t}\right)}_{\text{target}}-{\left(\text{t}\right)}_{\text{housekeeping gene}}\\ \Delta \text{C }{\left(\text{t}\right)}_{\text{calibrator}}=\text{ C }{\left(\text{t}\right)}_{\text{target}}-{\left(\text{t}\right)}_{\text{housekeeping gene}} \end{array}$$

The ΔΔC is then determined using the following formula:

$$\Delta \Delta \text{C }\left(\text{t}\right)=\;\Delta \text{C }{\left(\text{t}\right)}_{\text{sample}}-\Delta \text{C}{\left(\text{t}\right)}_{\text{calibrator}}$$

Expression of the target gene normalized to the housekeeping gene and relative to the calibrator = 2^-ΔΔC (t)^.

### Statistical analysis

The data analyses were performed using an SPSS version 12 (SPSS Inc, Chicago, IL, USA) software program. All data are expressed as mean ± SD. Statistical analysis was performed using post hoc tests in ANOVA; Differences between treatments were considered to be statistically significant at p < 0.05.

## Results

### The phospholipids fatty acid composition of cell membrane of HepG2 cell after the treatment of n-3 PUFA

When compared with the control group, the concentration of 22:6n-3, 20:5n-3, 18:3n-3 were significantly increased in the three treated groups respectively (p < 0.05); the level of n-3 PUFA was also significantly elevated in the three groups (p < 0.05) (Table 2).

**Table 2:** The fatty acid composition of cell membrane of HepG2 cell (%).

Fatty acid	Control	DHA	EPA	ALA
Total SFA	7.5 ± 1.1	7.5 ± 1.0	7.8 ± 1.2	7.6 ± 1.0
Total MUFA	75.3 ± 2.7	75.3 ± 3.1	74.5 ± 3.8	74.2 ± 2.9
Total n-6 PUFA	13.4 ± 1.3	12.5 ± 1.9	13.4 ± 2.0	13.4 ± 1.9
18:3n-3	1.3 ± 0.1^b^	1.3 ± 0.4^b^	1.5 ± 0.2^b^	2.4 ± 0.1^a^
20:5n-3	1.3 ± 0.2^b^	1.3 ± 0.1^b^	1.6 ± 0.4^a^	1.4 ± 0.0^b^
22:5n-3	0.4 ± 0.0	0.4 ± 0.2	0.5 ± 0.1	0.5 ± 0.0
22:6n-3	0.8 ± 0.2^b^	1.7 ± 1.0^a^	0.7 ± 0.1^b^	0.8 ± 0.3^b^
Total n-3 PUFA	3.8 ± 1.2^b^	4.7 ± 1.7^a^	4.3 ± 1.2^a^	5.1 ± 0.9^a^

The data was presented as Mean ± SD.

a, b, c Values within one row with different letters are significantly different (p < 0.5).

**SFA**: saturated fatty acid. **MUFA**: monounsaturated fatty acid. **PUFA**: polyunsaturated fatty acid. **DHA**: docosahexaenoic acid. **EPA**: eicosapentaenoic acid. **ALA**: alpha-linolenic acid.

### The mRNA expression of genes encoding the critical enzymes involved in methionine metabolism in HepG2 cell was determined by Real Time-PCR after the treatment of n-3 PUFA

**Figure 2:**
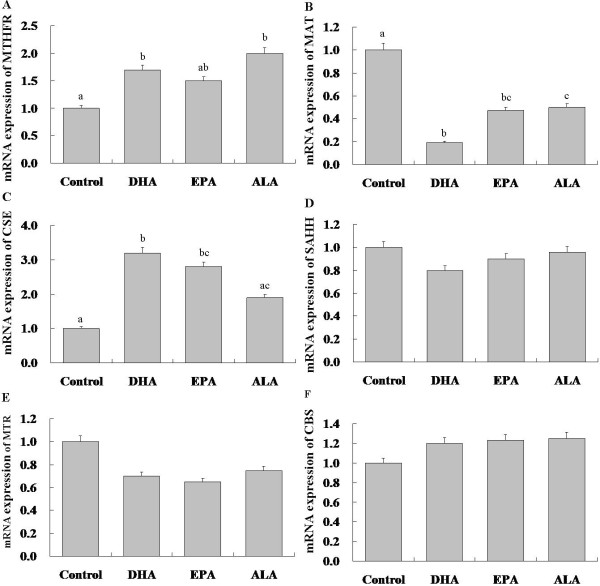
**The mRNA expression of genes in methionine metabolism in HepG-2 cell as determined by Real Time-PCR**. a, b, c Values within the four bars with different letters are significantly different (p < 0.5) (n = 9).

The expression levels of MTHFR were significantly increased in the DHA group (p < 0.05) and the ALA group (p < 0.05) when compared with control (Figure 2A);

Significantly decreased expression of MAT was observed in the three groups compared with control; furthermore, the level of MAT expression was significant lower in the DHA group than the ALA group (p < 0.05) (Figure 2B).

CSE expression was significantly increased in the DHA (p < 0.05) and EPA groups (p < 0.05) compared with control (Figure 2C).

No significant changes were seen in expression levels of SAHH, CBS, and MTR between the four groups (Figure 2D, E, F).

## Discussion

The objective of the present study was to investigate the regulatory effect of n-3 PUFA on mRNA expression of the genes encoding the critical enzymes involved in methionine metabolism.

We observed that n-3 PUFA up-regulates CSE and MTHFR mRNA expression and down-regulate MAT mRNA expression involved in Hcy metabolism. We suggest that this regulatory effect on gene expression is associated with decreased Hcy concentration.

A higher consumption of fish oil rich in n-3 PUFA is associated with reduced risk of cardiovascular events. Our previous studies have indicated that increased 22:6n-3 and n-3/n-6 PUFA in platelet and plasma PL is associated with decreased plasma Hcy [13,14]. Furthermore, meta-analysis confirms that n-3 PUFA decrease plasma Hcy [15]. In a previous animal study we have also demonstrated that 8 weeks of tuna oil treatment significantly decreases the plasma Hcy concentration in rats [24]. Yet HHcy is caused partly by genetic factors, including polymorphisms of genes encoding enzymes involved in Hcy metabolism, such as MTHFR, MTR, MTRR, and CBS [25,26]. A common mutation in MTHFR, MAT, and MTR results in a thermolabile variant with reduced activity [27,28]. Regulation of the gene expression of these critical genes using nutrients could make beneficial their coded enzyme activity.

To explain why n-3 PUFA decreases the level of Hcy, we examined the effect of n-3 PUFA on the mRNA expression of genes encoding the critical enzymes involved in methionine metabolism. In the present study, n-3 PUFA has been successfully incorporated into phospholipids of HepG2 cell membranes. Over the past 10 years, it has become evident that n-3 PUFA have a wide range of functions and are essential components of cells to maintain various functions and organelle structures, They can act as signaling molecules to regulate gene expression, encoding proteins forroles in fatty acid transport or metabolism, and act in differentiation, growth and metabolism [29,30]. Therefore, it is not surprising that the changes in PL fatty acid composition of cell membrane affected in the present studies can account for changes in mRNA expression of the critical genes involved in methionine metabolism, albeit with some selectivity.

We found that mRNA expression of MAT was significantly down-regulated by n-3PUFA. Thus, n-3 PUFA can affect the rate of SAM synthesis based on the activity of MAT. The intracellular SAM concentration is an important determinant of the fate of Hcy molecules [5]. But the resultant decrease in SAM synthesis via MAT would not stimulate SAH production which will be catalyzed to Hcy. The lack of recognizable change in SAHH expression presumably helps avoid an increase in Hcy, because the hydrolysis of SAH by the enzyme SAHH has been shown to be the sole intracellular source of Hcy [31]. SAHH expression was unchanged with n-3 PUFA when compared with control. Therefore, a decrease in SAH synthesis resulting from a decreased level of SAM would contribute to a reduction of Hcy formation.

SAM concentration plays an important role in the fate of Hcy molecules [5]. The potential mechanism is that SAM acts as not only as an allosteric inhibitor of MTHFR, but also an activator of CBS [32,33]. As such an effector, SAM suppresses the synthesis of N-5-methyltetrahydrofolate which is an important substrate required for remethylation and promotes the initial reaction of transsulfuration (CBS). Hcy, when synthesized, acquires a methyl group from N-5-methyltetrahydrofolate or from betaine to form methionine via vitamin B_12 _dependent MTHFR and MTR respectively in the remethylation pathway [5]. The impairment of the remethylation pathway due to an inadequate status of either folate or vitaminB_12 _or to defects in the gene encoding for MTHFR will lead to a substantial increase in plasma Hcy concentration [34,35]. In the present study, the MTR expression was not significantly affected. However, MAT was down-regulated by DHA, EPA, ALA; MTHFR was up-regulated by DHA and ALA when compared with control. Therefore, the decreased SAM concentration is insufficient for the inhibition of MTHFR; the result is an increased rate of N-5-methyltetrahydrofolate synthesis and Hcy remethylation [5], thereby reducing the level of Hcy.

In the transsulfuration pathway, Hcy condenses with serine to form cystathionine via CBS (using vitamin B_6 _as cofactor). Impairment in the transsulfuration pathway due to heterozygous defects in the CBS gene or inadequate levels of vitamin B_6 _will lead to a very slight increase in fasting plasma Hcy levels [36,37]. CBS and CSE are the most important determinants in the transsulfuration pathway. The present study did not show a significant effect of n-3 PUFA on mRNA expression of CBS, However, the CSE mRNA expression was significantly up-regulated by DHA and EPA. CSE, which is a vitamin B_6 _dependent enzyme, catalyzes the conversion of cystathionine into cysteine, α-ketobutyrate, taurine and H_2_S and is the rate-limiting enzyme for the synthesis of cysteine from Hcy [38]. In a previous study, we also found that the enzyme activity and mRNA expression of CSE was up-regulated after 8 weeks of tuna oil supplementation [24]. Thus up-regulated CSE mRNA expression expedited the degradation of cystathionine. This means that the transsulfuration reaction will move in a direction beneficial for a decrease in Hcy. Recently, elevated H_2_S has been proposed as a new gasotransmitter in the modulation of cardiovascular function [39,40]. Hcy-H_2_S metabolic imbalance could be an important mechanism in the pathogenesis of hypertension [40]. The up-regulated endogenous H_2_S/CSE pathway in pulmonary arteries by l-arginine is involved in the mechanisms by which l-arginine influences pulmonary hypertension [41]. The present findings also suggest that n-3 PUFA may ameliorate hypertension by decreasing Hcy as well as by increasing H_2_S as a result of up-regulated CSE gene expression.

The potential mechanisms by which n-3 PUFA regulate CSE, MAT, and MTHFR gene expression are not clear. Previous studies have demonstrated that n-3 PUFA governs oxidative gene expression involved in lipid metabolism by activating the transcription factor peroxisome proliferator-activated receptor (PPAR) alpha. N-3 PUFA suppress lipogenic gene expression by reducing the nuclear abundance and DNA-binding affinity of transcription factors responsible for imparting insulin and carbohydrate control to lipogenic and glycolytic genes. In particular, n-3 PUFA suppress the nuclear abundance and expression of sterol regulatory element binding protein-1 and reduce the DNA-binding activities of nuclear factor Y, Sp1 and possibly hepatic nuclear factor-4 [30]. Furthermore, Narayanan et al reported that DHA regulates genes and transcription factors in human colon cancer cells [42]; they showed that DHA down regulates nine members of the RNA II polymerases, transcription co-repressor associated protein and enhancer binding proteins such as AP2, in addition to changes in the expression of the zinc finger group of transcription factors and also altered expression of peroxisome proliferators (PPAR alpha and gamma) [42]. Based on these data, we speculate that a cis-acting n-3 PUFA responsive element (n-3 PUFA-RE) may be located in the promoter region of the n-3 PUFA-regulated genes. To alter gene transcription, a transcription factor (putative n-3 PUFA-binding protein) could bind to n-3 PUFA-RE and block or enhance transcription. In regard to this hypothesis, we found that the predicted transcription factors (RXR-alpha) bind to n-3 PUFA-RE in the promoter of MTHFR, CSE, and MAT though use of web based software Mapper: http://genome.ufl.edu/mapper/ (Table 3). Our hypothesis warrants further investigation.

**Table 3:** The predicted transcription factors binding to fatty acid-responsive element in the promoter of the gene involved in Hcy metabolism

Gene	GeneID	Transcript	Factor	Name(s)	Chrom	Start	End
MTR	4548	NM_000254	MA0066	PPARG	chr1	236,956,831	236,956,850
MTR	4548	NM_000254	MA0061	NF-kappaB	chr1	236,958,560	236,958,569
HHCY	191	NM_000687	MA0061	NF-kappaB	chr20	32891257	32891266
HHCY	191	NM_000687	M00242	PPARalpha:RXRalpha	chr20	32891807	32891826
HHCY	191	NM_000687	M00964	PXR, CAR, LXR, FXR	chr20	32892802	32892813
HHCY	191	NM_000687	T00720	RAR-gamma	chr20	32891787	32891797
HHCY	191	NM_000687	MA0074	RXRA::VDR	chr20	32891381	32891395
CSE	1491	NM_153742	M00774	NF-kappaB	chr1	70876211	70876222
CSE	1491	NM_153742	M00762	PPAR, HNF-4, COUP,	chr1	70875793	70875805
CSE	1491	NM_153742	M00518	PPARalpha:RXRalpha	chr1	70876539	70876552
CSE	1491	NM_153742	MA0065	PPARG::RXRA	chr1	70876537	70876553
CSE	1491	NM_153742	MA0074	RXRA::VDR	chr1	70875356	70875369
CSE	1491	NM_153742	T01331	RXR-alpha	chr1	70876645	70876657
CBS	875	NM_000071	MA0061	NF-kappaB	chr21	44496816	44496825
CBS	875	NM_000071	T00694	PPAR-alpha	chr21	44496841	44496851
CBS	875	NM_000071	MA0073	RREB1	chr21	44496183	44496202
CBS	875	NM_000071	MA0074	RXRA::VDR	chr21	44496362	44496376
CBS	875	NM_000071	T01349	RXR-beta	chr21	44496925	44496932
MAT1A	4143	NM_000429	T05257	CAR2:RXR-alpha	chr10	82051245	82051257
MAT1A	4143	NM_000429	M00631	FXR/RXR-alpha	chr10	82050669	82050682
MAT1A	4143	NM_000429	MA0061	NF-kappaB	chr10	82049620	82049629
MAT1A	4143	NM_000429	MA0105	NFKB1	chr10	82049504	82049514
MAT1A	4143	NM_000429	T02529	PPAR-gamma1	chr10	82051368	82051378
MAT1A	4143	NM_000429	MA0074	RXRA::VDR	chr10	82050026	82050040
MAT1A	4143	NM_000429	T01331	RXR-alpha	chr10	82050924	82050936
MTHFR	4524	NM_005957	MA0066	PPARG	chr1	11866220	11866239
MTHFR	4524	NM_005957	T05313	FXR:RXR-alpha	chr1	11867028	11867042
MTHFR	4524	NM_005957	M00774	NF-kappaB	chr1	11866612	11866623
MTHFR	4524	NM_005957	M00518	PPARalpha:RXRalpha	chr1	11867952	11867966
MTHFR	4524	NM_005957	MA0065	PPARG::RXRA	chr1	11867331	11867347
MTHFR	4524	NM_005957	T02529	PPAR-gamma1	chr1	11867417	11867427
MTHFR	4524	NM_005957	M00964	PXR, CAR, LXR, FXR	chr1	11866787	11866798

The predicted transcription factors (RXR-alpha) binding to n-3 PUFA-RE in the promoter of MTHFR, CSE, and MAT using web based software Mapper: http://genome.ufl.edu/mapper/.

## Conclusions

N-3 PUFA up-regulates CSE and MTHFR mRNA expression and down-regulates MAT mRNA expression involved in Hcy metabolism. This regulatory effect of n-3 PUFA on critical gene expression is associated with decreased Hcy concentration. Our findings provide a basis for verification of mechanisms by which n-3 PUFA decreases plasma Hcy.

## List of Abbreviations

SAHH: S-adenosylhomocysteine hydrolase; CBS: cystathionine β-synthase; CSE: cystathionine γ-lyase; MAT: methionine adenosyltransferase (S-adenosylmethionine synthase; methionine activating enzyme); MTR: 5-methyltetrahydrofolate-homocysteine methyltransferase; MTHFR: methylenetetrahydrofolate reductase; SAM: S-adenosylmethionine; SAH: S-adenosylhomocysteine; THF: tetrahydrofolate.

## Competing interests

The authors declare that they have no competing interests.

## Authors' contributions

TH carried out the studies, analyzed data and drafted the manuscript; MLW and DL participated in manuscript preparation; and MLW and DL participated in the project design. All authors read and approved the final manuscript.
